# Biomarkers in neurological disorders: a fast-growing market

**DOI:** 10.1093/braincomms/fcab086

**Published:** 2021-05-05

**Authors:** Alberto Lleó

**Affiliations:** Memory Unit, Neurology Department, Hospital de Sant Pau, Barcelona

In recent decades, biomarkers have been progressively incorporated in clinical routine and clinical trials in the field of neurology. The pace of discovery has been accelerating due to technological advances with increased precision and sensitivity of the instruments and assays, and cross-fertilization from one field of neurology to another. The arsenal of biomarkers in neurology is likely to keep growing as our ability to measure accurately multiple biological variables and our knowledge about the pathophysiology of the neurological diseases increase.

Biomarkers can be used for several purposes: to guide clinical diagnosis, to estimate disease risk or prognosis, to evaluate disease stage and to monitor progression or response to therapy.^1^ In clinical trials, biomarkers can be used to select a specific diagnostic subgroup (patient enrichment or stratification), to ensure appropriate therapeutic target engagement, to identify downstream effects of therapeutics on the disease process and as a measure of clinical efficacy and/or safety.^2^ Biomarkers are not new and the first descriptions can be found in the medical literature centuries ago. In 1611, the Italian philosophist and physician Santorio Sanctorius (1561–1636) created the first thermometer to measure body temperature. The creation of the thermometer was based on the thermoscope of Galileo Galilei. However, Santorio used the artefact to measure the temperature of a human body instead of the external temperature. This way Santorio’s instrument could be applied to monitor a biomarker of infection. Another device created by Santorio is the pulsilogium, aimed at measuring the heart frequency. By measuring biological processes objectively, Santorio made key contributions to the foundations of biomarkers and precision medicine.^3^

In recent decades, the discovery and implementation of biomarkers for neurological diseases have revolutionized the entire field. In 1995, Motter et al.^4^ published for the first time that patients with Alzheimer’s disease had low levels of amyloid-β_42_ in CSF. It took more than a decade to implement core CSF biomarkers (Aβ_42/40_, total tau and phosphorylated tau) in clinical routine, and many efforts are still ongoing to achieve a wider implementation. A key aspect of the implementation of a biomarker is harmonization of the procedure and development of certified reference material and methods.^2^^,^^5^ In multiple sclerosis, CSF oligoclonal bands were recently included in the revised diagnostic criteria.^6^ The excellent outcome of CSF biomarkers in Alzheimer’s disease has influenced positively other neurodegenerative diseases, and several CSF biomarkers are currently being investigated in Parkinson’s disease, frontotemporal dementia or dementia with Lewy bodies. While the first CSF assays relied on conventional immunoassays, the new protein aggregation assays initially applied to prion diseases, provide new opportunities in diseases with tau, TAR DNA binding protein-43 and α-synuclein-related pathologies.^7^ It is plausible that these assays could be also incorporated in the near future in the diagnosis of α-synucleinopathies and other disorders needed of specific pathophysiological biomarkers.

A major advance in the field of neurology has been the development of blood-based biomarkers. Despite initial scepticism in peripheral markers due to the physical restrictions imposed by the blood brain barrier, recent technological advances have made possible to measure analytes in different biofluids in very low concentrations. The new instruments are mostly based on Simoa or Mass spectrometry, which provide an optimal analytical sensitivity. A key advance in the field is the possibility to measure neurofilaments in blood as a measure of neuronal damage in a wide range of neurological conditions, such as neurodegenerative disorders, multiple sclerosis, traumatic brain injury, peripheral neuropathies or COVID-19 neurological-associated damage.^8^^,^^9^ The possibility to measure CNS markers in an easily accessible source, provides a window to use blood markers to track disease progression in clinical routine or in clinical trials. For example, blood neurofilament light protein (NfL) concentrations can be useful to monitor disease progression in multiple sclerosis or brain damage after cardiac arrest or traumatic brain injury, among many other applications.^8^ In addition to NfL, there are many other blood analytes with potential clinical application. Recently, assays to measure different isoforms of phosphorylated tau have been described.^10^ All these assays can detect Alzheimer’s disease pathology with high accuracy, and they are expected to move quickly towards clinical routine.

Finally, we have observed notable advances in imaging markers in neurology. Novel MRI modalities have been key in several neurological conditions to analyse subtle changes in brain macro- and microstructure. New seven Tesla-MRI scanners can provide high resolution for detecting nearly microscopic changes in different neurological conditions. An important milestone in imaging research was the ability to develop radiotracers that can bind protein aggregates in the brain. Amyloid positron emission tomography using Pittsburgh compound B was the first to be developed,^11^ and later other ^18^F-based radiotracers have followed the steps. These radiotracers are currently approved in many countries for the diagnosis of Alzheimer’s disease. Tau tracers were developed latter and are being applied to Alzheimer’s disease, chronic traumatic encephalopathy and other tauopathies.^12^ Other tracers able to track synaptic loss or inflammatory processes show high promise for many neurological conditions.

In the current special collection of Brain Communications on biomarkers, the journal has gathered a sample of relevant papers under this topic. The collection includes articles from blood biomarkers to novel EEG or MRI metrics in several neurological conditions. Many studies aim to predict disease progression using baseline imaging or fluid biomarkers, others look at longitudinal trajectories as surrogate measures of clinical course.

In years to come, we will see new additional exciting biomarkers that will allow detection of neurological diseases at early disease stages and simultaneous monitoring of multiple biological pathways in response to sophisticated therapeutic interventions. We have to prepare for the fascinating era of precision medicine, an era initiated by Santorio, an era the limits of which he could never have imagined.

## Data availability

Data sharing is not applicable to this article as no new data were created or analysed.

## Competing interests

A.L. has served as a consultant or at advisory boards for Fujirebio-Europe, Roche Diagnostics, Biogen and Nutricia. In addition, A.L. has a patent WO2019175379 A1 Markers of synaptopathy in neurodegenerative disease issued.
